# Macroscopic Quantum-Type Potentials in Theoretical Systems Biology

**DOI:** 10.3390/cells3010001

**Published:** 2013-12-30

**Authors:** Laurent Nottale

**Affiliations:** CNRS, LUTH, Paris Observatory and Paris-Diderot University, Meudon Cedex 92195, France; E-Mail: laurent.nottale@obspm.fr; Tel.: +33-145-077-403

**Keywords:** systems biology, relativity, fractals

## Abstract

We review in this paper the use of the theory of scale relativity and fractal space-time as a tool particularly well adapted to the possible development of a future genuine systems theoretical biology. We emphasize in particular the concept of quantum-type potentials, since, in many situations, the effect of the fractality of space—or of the underlying medium—can be reduced to the addition of such a potential energy to the classical equations of motion. Various equivalent representations—geodesic, quantum-like, fluid mechanical, stochastic—of these equations are given, as well as several forms of generalized quantum potentials. Examples of their possible intervention in high critical temperature superconductivity and in turbulence are also described, since some biological processes may be similar in some aspects to these physical phenomena. These potential extra energy contributions could have emerged in biology from the very fractal nature of the medium, or from an evolutive advantage, since they involve spontaneous properties of self-organization, morphogenesis, structuration and multi-scale integration. Finally, some examples of applications of the theory to actual biological-like processes and functions are also provided.

## 1. Introduction

The theory of scale relativity and fractal space-time accounts for a possibly nondifferentiable geometry of the space-time continuum, based on an extension of the principle of relativity to scale transformations of the reference system. Its framework was revealed to be particularly well adapted to a new theoretical approach of systems biology [1,2,3].

This theory was initially built with the goal of re-founding quantum mechanics on prime principles [4,5,6]. The success of this enterprise [7,8] has been completed by obtaining new results: in particular, a generalization of standard quantum mechanics at high energy to new forms of scale laws [9], and the discovery of the possibility of macroscopic quantum-type behavior under certain conditions [10], which may well be achieved in living systems.

This new “macroquantum” mechanics (or “mesoquantum” at, e.g., the cell scale) no longer rests on the microscopic Planck constant ℏ. The parameter, which replaces ℏ is specific to the system under consideration, emerges from self-organization of this system and can now be macroscopic or mesoscopic. This theory is specifically adapted to the description of multi-scale systems capable of spontaneous self-organization and structuration. Two privileged domains of applications are, therefore, astrophysics [6,8,10,11,12,13] and biophysics [1,2,8,14,15].

In this contribution dedicated to applications in biology, after a short reminder of the theory and of its methods and mathematical tools, we develop some aspects which may be relevant to its explicit use for effective biophysical problems. A special emphasis is placed on the concept of macroquantum potential energy. Scale relativity methods are relevant because they provide new mathematical tools to deal with scale-dependent fractal systems, like equations in scale space and scale-dependent derivatives in physical space. This approach is also very appropriate for the study of biological systems because its links micro-scale fractal structures with organized form at the level of an organism.

For more information the interested reader may consult the two detailed papers [1,2] and references therein.

## 2. Brief Reminder of the Theory

The theory of scale relativity consists of introducing, in an explicit way, the scale of measurement (or of observation) *ε* in the (bio-)physical description. These scale variables can be identified, in a theoretical framework, to the differential elements *ε* = *dX*, and, in an experimental or observational framework, to the resolution of the measurement apparatus.

The coordinates can now be explicit functions of these variables, *X* = *X*(*dX*) (we omit the indices for simplicity of writing, but the coordinates are in general vectors while the resolution variables are tensors [8], Chapter 3.6). In case of divergence of these functions toward small scales, they are fractal coordinates. The various quantities which describe the system under consideration become themselves fractal functions, *F* = *F*[*X*(*dX*), *dX*]. In the simplified case when the fractality of the system is but a consequence of that of space, there is no proper dependence of *F* in function of *dX*, and we have merely *F* = *F*[*X*(*dX*)].

The description of such an explicitly scale dependent system needs three levels instead of two. Usually, one makes a transformation of coordinates *X* → *X* + *dX*, then one looks for the effect of this infinitesimal transformation on the system properties, *F* → *F* + *dF*. This leads to write differential equations in terms of space-time coordinates.

However, in the new situation, since the coordinates are now scale dependent, one should first state the laws of scale transformation, *ε* → *ε′*, then their consequences on the coordinates, *X*(*ε*) → *X′*(*ε′*) and finally on the various (bio-)physical quantities *F*[*X*(*ε*)] → *F′* [*X′*(*ε′*)]. One of the main methods of the scale relativity theory consists of describing these scale transformations using differential equations playing in scale space (*i.e.*, the space of the scale variables {*ε*}). In other words, one considers infinitesimal scale transformations, ln(*ε*/λ) → ln(*ε*/λ) + *d*ln(*ε*/λ), rather than the discrete iterated transformations that have been most often used in the study of fractal objects [16,17,18].

The motion equations in scale relativity are therefore obtained in the framework of a double partial differential calculus acting both in space-time (positions and instants) and in scale space (resolutions), basing oneself on the constraints imposed by the double principle of relativity, of motion and of scale.

### 2.1. Laws of Scale Transformation

The simplest possible scale differential equation which determines the length of a fractal curve (*i.e.*, a fractal coordinate) 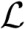 reads

(1) $$\frac{\partial \text{L}}{\partial \ln\varepsilon }=a+b\text{L}$$

where *∂*/*∂* ln *ε* is the dilation operator [8,9]. Its solution combines a self-similar fractal power-law behavior and a scale-independent contribution:

(2) $$\text{L}(\varepsilon )={\text{L}}_{0}\left\{1+{\left(\frac{\lambda }{\varepsilon }\right)}^{\tau_{F}}\right\}$$

where λ is an integration constant and where *τ_F_* = −*b* = *D_F_* − 1. One easily verifies that the fractal part of this expression agrees with the principle of relativity applied to scales. Indeed, under a transformation *ε* → *ε′*, it transforms as 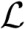 = 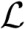
_0_(*ε′*/*ε*)*^τ_F_^* and therefore it depends only on the ratio between scales and not on the individual scales themselves.

This result indicates that, in a general way, fractal functions are the sum of a differentiable part and of a non-differentiable (fractal) part, and that a spontaneous transition is expected to occur between these two behaviors.

On the basis of this elementary solution, generalized scale laws can be naturally obtained by now considering second order differential equations in scale space. This is reminiscent of the jump from the law of inertial motion, *dX*/*dt* = *V* = *cst* to the fundamental law of dynamics *d*^2^*X*/*dt*^2^ = *F* as concerns motion. The same evolution can be suggested for scale laws: one can jump from scale invariance—possibly broken beyond some transition scale—described by first order differential scale equations, to a “scale dynamics”, involving “scale forces” and second order differential equations.

Many of these generalizations may be relevant in biology, in particular:

– log-periodic corrections to power laws:
  (3) $$\text{L}(\varepsilon )=a\varepsilon^{\nu }[1+b\cos(\omega \ln\varepsilon )]$$
  which is a solution of a second-order differential wave equation in scales.
– law of “scale dynamics” involving a constant “scale acceleration”:
  (4) $$\begin{array}{ll} \tau_{F}=\frac{1}{G}\ln\left(\frac{\lambda_{0}}{\varepsilon }\right), & \ln\left(\frac{\text{L}}{{\text{L}}_{0}}\right) \end{array}=\frac{1}{2G}\ln^{2}\left(\frac{\lambda_{0}}{\varepsilon }\right)$$
  This law may be the manifestation of a constant “scale force”, which describes the difference with the free self-similar case (in analogy with Newton's dynamics of motion). In this case the fractal dimension is no longer constant, but varies in a linear way in terms of the logarithm of resolution. Many manifestations of such a behavior have been identified in human and physical geography [19,20].
– law of “scale dynamics” involving a scale harmonic oscillator:
  (5) $$\ln\frac{\text{L}}{{\text{L}}_{0}}=\tau_{0}\sqrt{\ln^{2}\frac{\lambda_{0}}{\varepsilon }-\ln^{2}\frac{\lambda_{0}}{\lambda_{1}}}$$
  For *ε* ≪ λ_0_ it gives the standard scale invariant case 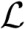 = 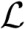
_0_(λ_0_/*ε*)^*τ*^0^^, *i.e.*, constant fractal dimension *D_F_* = 1 + *τ*_0_. But its intermediate-scale behavior is particularly interesting, since, owing to the form of the mathematical solution, resolutions larger than a scale λ_1_ are no longer possible. This new kind of transition therefore separates small scales from large scales, *i.e.*, an “interior” (scales smaller than λ_1_) from an “exterior” (scales larger than λ_1_). It is characterized by an effective fractal dimension that becomes formally infinite. This behavior may prove to be particularly interesting for applications to biology, as we shall see in Section 6.
– laws of special scale relativity [9]:
  (6) $$\ln\frac{\text{L}(\varepsilon )}{{\text{L}}_{0}}=\frac{\tau_{0}\ln(\lambda_{0}/\varepsilon )}{\sqrt{1-\ln^{2}(\lambda_{0}/\varepsilon )/\ln^{2}(\lambda_{0}/\lambda_{H})}}$$
  (7) $$\tau_{F}(\varepsilon )=\frac{\tau_{0}}{\sqrt{1-\ln^{2}(\lambda_{0}/\varepsilon )/\ln^{2}(\lambda_{0}/\lambda_{H})}}$$
  This case may not be fully relevant in biology, but we recall it here because it is one of the most profound manifestations of scale relativity. Here the length (*i.e.*, the fractal coordinate) and the ‘djinn’ (variable fractal dimension minus topological dimension) *τ_F_* = *D_F_* − 1 have become the components of a vector in scale space. In this new law of scale transformation, a limiting scale appears, λ*_H_*, which is impassable and invariant under dilations and contractions, independently of the reference scale λ_0_. We have identified this invariant scale to the Planck length $l_{\text{P}}=\sqrt{\hbar G/c^{3}}$ toward small scales, and to the cosmic length $\text{L}=1/\sqrt{\Lambda }$ (where Λ is the cosmological constant) toward large scales [6,8,9].

Many other scale laws can be constructed as expressions of Euler-Lagrange equations in scale space, which give the general form expected for these laws [8], Chapter 4.

### 2.2. Laws of Motion

The laws of motion in scale relativity are obtained by writing the fundamental equation of dynamics (which is equivalent to a geodesic equation in the absence of an exterior field) in a fractal space. The non-differentiability and the fractality of coordinates implies at least three consequences [6,8]:

(1) The number of possible paths is infinite. The description therefore naturally becomes non-deterministic and probabilistic. These virtual paths are identified with the geodesics of the fractal space. The ensemble of these paths constitutes a fluid of geodesics, which is therefore characterized by a velocity field.
(2) Each of these paths is itself fractal. The velocity field is therefore a fractal function, explicitly dependent on resolutions and divergent when the scale interval tends to zero (this divergence is the manifestation of non-differentiability).
(3) Moreover, the non-differentiability also implies a two-valuedness of this fractal function, (*V*_+_, *V*_−_). Indeed, two definitions of the velocity field now exist, which are no longer invariant under a transformation |*dt*| → −|*dt*| in the non-differentiable case.

These three properties of motion in a fractal space lead to describing the geodesic velocity field in terms of a complex fractal function 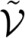 = (*V*_+_ + *V*_−_)/2 − *i*(*V*_+_ − *V*_−_)/2. The (+) and (−) velocity fields can themselves be decomposed in terms of a differentiable part *υ*_±_ and of a fractal (divergent) fluctuation of zero mean *w*_±_, *i.e.*, *V*_±_ = *υ*_±_ + *w*_±_ and therefore the same is true of the full complex velocity field, 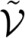 = 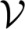
(*x*, *y*, *z*, *t*) + 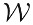
(*x*, *y*, *z*, *t*, *dt*).

Jumping to elementary displacements along these geodesics, this reads *dX*_±_ = *d*_±_*x* + *dξ*_±_, with (in the case of a critical fractal dimension *D_F_* = 2 for the geodesics)

(8) $$\begin{array}{ll} d_{\pm }x=\upsilon_{\pm }\;dt, & d\xi_{\pm }=\zeta_{\pm }\sqrt{2D}{\left|dt\right|}^{1/2} \end{array}$$

This case is particularly relevant since it corresponds to a Markov-like situation of loss of information from one point to the following, without correlation nor anti-correlation. Here ζ_±_ represents a dimensionless stochastic variable such that <ζ_±_> = 0 and $<\zeta_{\pm }^{2}>=1$. The parameter 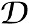 characterizes the amplitude of fractal fluctuations.

These various effects can be combined under the construction of a total derivative operator [6] :

(9) $$\frac{\hat{d}}{dt}=\frac{\partial }{\partial t}+\text{V}\cdot \nabla -i\text{D}\Delta$$

The fundamental equation of dynamics becomes, in terms of this operator

(10) $$m\frac{\hat{d}}{dt}\text{V}=-\nabla \phi$$

In the absence of an exterior field *ϕ*, this is a geodesic equation (*i.e.*, a free inertial Galilean-type equation).

The next step consists of making a change of variable in which one connects the velocity field 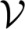 = *V* − *iU* to a function *ψ* according to the relation

(11) $$\text{V}=-i\frac{S_{0}}{m}\nabla \ln\psi$$

The parameter *S*_0_ is a constant for the system considered (it identifies to the Planck constant ℏ in standard quantum mechanics). Thanks to this change of variable, the equation of motion can be integrated under the form of a Schrödinger equation [6,8] generalized to a constant different from ℏ,

(12) $${\text{D}}^{2}\Delta \psi +i\text{D}\frac{\partial }{\partial t}\psi -\frac{\phi }{2m}\psi =0$$

where the two parameters introduced above, *S*_0_ and 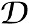, are linked by the relation:

(13) $$S_{0}=2m\text{D}$$

In the case of standard quantum mechanics, *S*_0_ = ℏ, so that 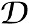 is a generalization of the Compton length (up to the constant *c*) and Equation (13) is a generalization of the Compton relation

(14) $$\lambda_{C}=\frac{2\text{D}}{c}=\frac{\hbar }{mc}$$

We obtain the same result by using the full velocity field including the fractal fluctuations of zero mean [8]. This implies the possible existence of fractal solutions for quantum mechanical equations [21,22].

By setting finally $\psi =\sqrt{P}\times e^{i\theta }$, with *V* = 2 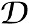
∇*θ*, one can show (see [7,8] and next section) that *P* = |*ψ*|^2^ gives the number density of virtual geodesics. This function becomes naturally a density of probability, or a density of matter or radiation, according to the various conditions of an actual experiment (one particle, many particles or a radiation flow). The function *ψ*, being solution of the Schrödinger equation and subjected to the Born postulate and to the Compton relation, owns therefore most of the properties of a wave function.

Reversely, the density *ρ* and the velocity field *V* of a fluid in potential motion can be combined in terms of a complex function $\psi =\sqrt{\rho }\times e^{i\theta }$ which may become a wave function solution of a Schrödinger equation under some conditions, in particular in the presence of a quantum-type potential (see next section).

## 3. Multiple Representations

After this brief summary of the theory (see more details in [8]), let us now consider some of its aspects that may be particularly relevant to applications in biology. One of them is the multiplicity of equivalent representations of the same equations. Usually, classical deterministic equations, quantum equations, stochastic equations, fluid mechanics equations, *etc.* correspond to different systems and even to different physical laws. But in the scale relativity framework, they are unified as being different representations of the same fundamental equation (the geodesic equation of relativity), subjected to various changes of variable. This is a particularly useful tool in biophysics, which makes often use of diffusion equations of the Fokker-Planck type or of fluid mechanics equations.

### 3.1. Geodesic Representation

The first representation, which can be considered as the root representation, is the geodesic one. The two-valuedness of the velocity field is expressed in this case in terms of the complex velocity field 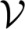 = *V* − *iU*. It implements what makes the essence of the principle of relativity, *i.e.*, the equation of motion must express the fact that any motion should disappear in the proper system of coordinates:

(15) V=0

By deriving this equation with respect to time, it takes the form of a free inertial equation devoid of any force:

(16) $$\frac{\hat{d}}{dt}\text{V}=0$$

where the “covariant” derivative operator *d̂*/*dt* includes the terms which account for the effects of the geometry of space-(time). In the case of a fractal space, it reads as we have seen

(17) $$\frac{\hat{d}}{\partial t}=\frac{\partial }{\partial t}+\text{V}\cdot \nabla -i\text{D}\Delta$$

### 3.2. Quantum-Type Representation

We have recalled in the previous section how a wave function *ψ* can be introduced from the velocity field of geodesics:

(18) V=−2iD∇lnψ

This mean that the doubling of the velocity field issued from non-differentiability is expressed in this case in terms of the modulus and the phase of this wave function. This allows integration of the equation of motion in the form of a Schrödinger equation,

(19) $${\text{D}}^{2}\Delta \psi +i\text{D}\frac{\partial }{\partial t}\psi -\frac{\phi }{2m}\psi =0$$

By making explicit the modulus and the phase of the wave function, $\psi =\sqrt{P}\times e^{i\theta }$, where the phase is related to the classical velocity field by the relation *V* = *2* 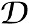
∇*θ*, one can give this equation the form of hydrodynamics equations including a quantum potential. Moreover, it has been recently shown that this transformation is reversible, *i.e.*, by adding a quantum-like potential energy to a classical fluid, it becomes described by a Schrödinger equation and therefore acquires some quantum-type properties [8,23].

### 3.3. Fluid Representation with Macroquantum Potential

It is also possible, as we shall now see, to go directly from the geodesic representation to the fluid representation without writing the Schrödinger equation.

To this purpose, let us express the complex velocity field in terms of the classical (real) velocity field *V* and of the number density of geodesics *P_N_*, which is equivalent as we have seen above to a probability density *P*:

(20) V=V−iD∇lnP

The quantum covariant derivative operator thus reads

(21) $$\frac{\hat{d}}{\partial t}=\frac{\partial }{\partial t}+V.\nabla -i\text{D}(\nabla \ln P.\nabla +\Delta )$$

The fundamental equation of dynamics becomes (introducing also an exterior scalar potential *ϕ*):

(22) $$\left(\frac{\partial }{\partial t}+V.\nabla -i\text{D}(\nabla \ln P.\nabla +\Delta )\right)(V-i\text{D}\nabla \ln P)=-\frac{\nabla \phi }{m}$$

The imaginary part of this equation,

(23) $$\text{D}\left\{(\nabla \ln P.\nabla +\Delta )V+\left(\frac{\partial }{\partial t}+V.\nabla \right)\nabla \ln P\right\}=0$$

takes, after some calculations, the following form

(24) $$\nabla \left\{\frac{1}{P}\left(\frac{\partial }{\partial t}+\text{div}(PV)\right)\right\}=0$$

and it can finally be integrated in terms of a continuity equation

(25) $$\frac{\partial P}{\partial t}+\text{div}(PV)=0$$

The real part,

(26) $$\left(\frac{\partial }{\partial t}+V.\nabla \right)V=-\frac{\nabla \phi }{m}+{\text{D}}^{2}(\nabla \ln P.\nabla +\Delta )\nabla \ln P$$

takes the form of an Euler equation,

(27) $$m\left(\frac{\partial }{\partial t}+V.\nabla \right)V=-\nabla \phi +2m{\text{D}}^{2}\nabla \left(\frac{\Delta \sqrt{P}}{\sqrt{P}}\right)$$

and it therefore describes a fluid subjected to an additional quantum-type potential

(28) $$Q=-2m{\text{D}}^{2}\frac{\Delta \sqrt{P}}{\sqrt{P}}$$

It is remarkable that we have obtained this result directly, without passing through a quantum-type representation using a wave function nor through a Schrödinger equation.

The additional “fractal” potential is obtained here as a mere manifestation of the fractal geometry of space, in analogy with Newton's potential emerging as a manifestation of the curved geometry of space-time in Einstein's relativistic theory of gravitation. We have suggested ([8] and references therein) that this geometric energy could contribute to the effects which have been attributed in astrophysics to a missing “dark matter” (knowing that all attempts to directly observe this missing mass have so far failed). Another suggestion, relevant to biology, is that such a potential energy could play an important role in the self-organization and in the morphogenesis of living systems [2,24].

### 3.4. Coupled Two-Fluids

Another equivalent possible representation consists of separating the real and imaginary parts of the complex velocity field,

(29) V=V−iU

One obtains in this case a system of equations that describe the velocity fields of two fluids strongly coupled together,

(30) $$\left(\frac{\partial }{\partial t}+V.\nabla \right)V=(U.\nabla +\text{D}\Delta )U-\nabla \left(\frac{\phi }{m}\right)$$

(31) $$\left(\frac{\partial }{\partial t}+V.\nabla \right)U=-(U.\nabla +\text{D}\Delta )V$$

This representation may be useful in, e.g., numerical simulations of scale relativity/quantum processes [25].

### 3.5. Diffusion-Type Representation

The fundamental two-valuedness which is a consequence of non-differentiability has been initially described in terms of two mean velocity fields *υ*_+_ and *υ*_−_, which transform one into the other by the reflexion |*dt*| ↔ − |*dt*|. It is therefore possible to write the equations of motion directly in terms of these two velocity fields. The representation obtained in this way implements the diffusive character of a fractal space and is therefore particularly interesting for biophysical applications. Indeed, one obtains the standard Fokker-Planck equation for the velocity *υ*_+_, as for a classical stochastic process:

(32) $$\frac{\partial P}{\partial t}+\text{div}(P\upsilon_{+})=\text{D}\Delta P$$

where the parameter 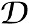 plays the role of a diffusion coefficient. On the contrary, the equation obtained for the velocity field *υ*_−_ does not correspond to any classical process:

(33) $$\frac{\partial P}{\partial t}+\text{div}(P\upsilon_{-})=-\text{D}\Delta P$$

This equation is derived from the geodesic equation on the basis of non-differentiability, but it cannot be set as a founding equation in the framework of a standard diffusion process as was proposed by Nelson [26], since it becomes self-contradictory with the backward Kolmogorov equation generated by such a classical process [10,27,28] and [8], (p. 384).

### 3.6. A New Form of Quantum-Type Potential

However, one may remark that the previous representation is not fully coherent, since it involves three quantities *P*, *υ*_+_ and *υ*_−_ instead of two expected from the velocity doubling. Therefore it should be possible to obtain a system of equations involving only the probability density *P* and one of the velocity fields, here *υ*_+_. To this purpose, one remarks that *υ*_−_ is given in terms of these two quantities by the relation:

(34) $$\upsilon_{-}=\upsilon_{+}-2\text{D}\nabla \ln P$$

We also recall that

(35) $$V=\upsilon_{+}-\text{D}\nabla \ln P$$

The energy equation now reads

(36) $$E=\frac{1}{2}mV^{2}+Q+\phi =\frac{1}{2}m{\left(\upsilon_{+}-\text{D}\nabla \ln P\right)}^{2}+Q+\phi$$

where the macroquantum potential can be written

(37) $$Q=-2m{\text{D}}^{2}\frac{\Delta \sqrt{P}}{\sqrt{P}}=-m{\text{D}}^{2}\left\{\Delta \ln P+\frac{1}{2}{\left(\nabla \ln P\right)}^{2}\right\}$$

One of the terms of this “fractal potential” is therefore compensated while another term appears, so that we obtain:

(38) $$E=\frac{1}{2}m\upsilon_{+}^{2}+\phi -m\text{D}\upsilon_{+}.\nabla \ln P-m{\text{D}}^{2}\Delta \ln P$$

We finally obtain a new representation in terms of a Fokker-Planck equation, which contains the diffusive term 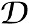
Δ*P* in addition to the continuity equation obtained in the case of the fluid representation (*V*, *P*), and an energy equation which includes a new form of quantum potential:

(39) $$\frac{\partial P}{\partial t}+\text{div}(P\upsilon_{+})=\text{D}\Delta P$$

(40) $$E=\frac{1}{2}m\upsilon_{+}^{2}+\phi +Q_{+}$$

where the new quantum-type potential reads

(41) $$Q_{+}=-m\text{D}(\upsilon_{+}.\nabla \ln P+\text{D}\Delta \ln P)$$

It now depends not only on the probability density *P*, but also on the velocity field *υ*_+_.

This derivation is once again reversible. This means that a classical diffusive system described by a standard Fokker-Planck equation which would be subjected to such a generalized quantum-type potential would be spontaneously transformed into a quantum-like system described by a Schrödinger Equation (19) acting on a wave function $\psi =\sqrt{P}\times e^{i\theta }$ where *V* = 2 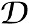
∇*θ*. Thanks to Equation (35), this wave function is defined in terms of *P* and *υ*_+_ as

(42) $$\psi ={\sqrt{P}}^{1-i}\times e^{i\theta_{+}}$$

where *υ*_+_ = 2 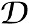
∇θ_+_.

Such a system, although it is initially diffusive, would therefore acquire some quantum-type properties, but evidently not all of them: the behaviors of coherence, inseparability, indistinguishability or entanglement are specific of a combination of quantum laws and elementarity [29] and cannot be recovered in such a context.

This is nevertheless a remarkable result, which means that a partial reversal of diffusion and a transformation of a classical diffusive system into a quantum-type self-organized one should be possible by applying a quantum-like force to this system. This is possible in an actual experiment consisting of a retro-active loop involving continuous measurements, not only of the density [23] but also of the velocity field *υ*_+_, followed by a real time application on the system of a classical force *F_Q_*_+_ = −∇*Q*_+_ simulating the new macroquantum force [30].

One may also wonder whether living systems, which already work in terms of such a feedback loop (involving sensors, then cognitive processes, then actuators) could have naturally included such kinds of quantum-like potentials in their operation through the selection/evolution process, simply because it provides an enormous evolutionary advantage due to its self-organization and morphogenesis negentropic capabilities [2] and ([8], Chapter 14).

### 3.7. Quantum Potential Reversal

One of the recently obtained results which may be particularly relevant to the understanding of living systems concerns the reversal of the quantum-type potential. What happens when the potential energy keeps exactly the same form, as given by $\Delta \sqrt{P}/\sqrt{P}$ for a given distribution *P*(*x*,*y*,*z*), while its sign is reversed ? In other words, to what kind of process does the equation

(43) $$\left(\frac{\partial }{\partial t}+V.\nabla \right)V=-\frac{\nabla \phi }{m}-2{\text{D}}^{2}\nabla \frac{\Delta \sqrt{P}}{\sqrt{P}}$$

correspond?

We have shown [2,8] that such an Euler equation, when it is combined with a continuity equation, can no longer be integrated under the form of a generalized Schrödinger equation. This process is therefore no longer self-organizing. On the contrary, this is a classical diffusive process, characterized by an entropy increase proportional to time.

Indeed, let us start from a Fokker-Planck equation

(44) $$\frac{\partial P}{\partial t}+\text{div}(P\upsilon )=D\Delta P$$

which describes a classical diffusion process with diffusion coefficient *D*. Then make the change of variable

(45) V=υ−D∇lnP

One finds after some calculations that *V* and *P* are now solutions of a continuity equation

(46) $$\frac{\partial P}{\partial t}+\text{div}(P\upsilon )=0$$

and of an Euler equation which reads

(47) $$\left(\frac{\partial }{\partial t}+V.\nabla \right)V=-2D^{2}\nabla \frac{\Delta \sqrt{P}}{\sqrt{P}}$$

In other words, we have obtained an hydrodynamical description of a standard diffusion process in terms of a “diffusion potential” which is exactly the reverse of the macroquantum potential.

We have suggested that this behavior may be relevant for the understanding of cancer [2,8] (see also [31] about the relationship between fractal geometry and tumors), since a mere change of sign of the additional potential leads to dramatic consequences: the self-organizing, morphogenetic and structuring character of the system is instantaneously changed to a diffusive, anti-structuring disorganization.

## 4. Quantum Potentials in High-Temperature Superconductivity

### 4.1. Ginzburg-Landau Non-Linear Schrödinger Equation

The phenomenon of superconductivity is one of the most fascinating of physics. It lies at the heart of a large part of modern physics. Indeed, besides its proper interest for the understanding of condensed matter, it has been used as model for the construction of the electroweak theory through the Higgs field and of other theories in particle physics and in other sciences.

Moreover, superconductivity (SC) has led physicists to deep insights about the nature of matter. It has shown that the ancient view of matter as something “solid”, in other words “material”, was incorrect. The question: “is it possible to walk through walls” is now asked in a different way. Nowadays we know that it is not a property of matter by itself which provides it qualities such as solidity or ability to be crossed, but its interactions.

A first relation of SC with the scale relativity approach can be found in its phenomenological Ginzburg-Landau equation. Indeed, one can recover such a non-linear Schrödinger equation simply by adding a quantum-like potential energy to a standard fluid including a pressure term [23].

Consider indeed an Euler equation with a pressure term and a quantum potential term:

(48) $$\left(\frac{\partial }{\partial t}+V\cdot \nabla \right)V=-\nabla \phi -\frac{\nabla p}{\rho }+2{\text{D}}^{2}\nabla \left(\frac{\Delta \sqrt{\rho }}{\sqrt{\rho }}\right)$$

When ∇*p*/*ρ* = ∇*w* is itself a gradient, which is the case of an isentropic fluid, and, more generally, of every cases when there is a state equation which links *p* and *ρ*, its combination with the continuity equation can be still integrated in terms of a Schrödinger-type equation [10],

(49) $${\text{D}}^{2}\Delta \psi +i\text{D}\frac{\partial }{\partial t}\psi -\frac{\phi +w}{2}\psi =0$$

In the sound approximation, the link between pressure and density writes $p-p_{0}=c_{s}^{2}(\rho -\rho_{0})$, where *c_s_* is the sound speed in the fluid, so that $\nabla p/\rho =c_{s}^{2}\nabla \ln\rho$. Moreover, when *ρ* − *ρ*_0_ ≪ *ρ*_0_, one may use the additional approximation $c_{s}^{2}\nabla \ln\rho \approx (c_{s}^{2}/\rho_{0})\nabla \rho$, and the equation obtained takes the form of the Ginzburg-Landau equation of superconductivity [32],

(50) $${\text{D}}^{2}\Delta \psi +i\text{D}\frac{\partial }{\partial t}\psi -\beta {\left|\psi \right|}^{2}\psi =\frac{1}{2}\phi \psi$$

with $\beta =c_{s}^{2}/2\rho_{0}$. In the highly compressible case, the dominant pressure term is rather of the form *p* ∝ *ρ*^2^, so that *p*/*ρ* ∝ *ρ* = |*ψ*|^2^, and one still obtains a non-linear Schrodinger equation of the same kind [33].

The intervention of pressure is highly probable in living systems, so that such an equation is expected to be relevant in theoretical systems biology Laboratory experiments aiming at implementing this transformation of a classical fluid into a macroscopic quantum-type fluid are presently under development [30,34].

### 4.2. A Quantum Potential as Origin of Cooper Pairs in HTS?

Another important question concerning SC is that of the microscopic theory which gives rise to such a macroscopic phenomenological behavior.

In superconducting materials, the bounding of electrons in Cooper pairs transforms the electronic gas from a fermionic to a bosonic quantum fluid. The interaction of this fluid with the atoms of the SC material becomes so small that the conducting electrons do not “see” any longer the material. The SC electrons become almost free, all resistance is abolished and one passes from simple conduction to superconduction.

In normal superconductors, the pairing of electrons is a result of their interaction with phonons (see, e.g., [35]). But since 1985, a new form of superconductivity has been discovered which has been named “high temperature superconductivity” (HTS) because the critical temperature, which was of the order of a few kelvins for normal SC, has reached up to 135 K. However, though it has been shown that HTS is still due to the formation of Cooper pairs, the origin of the force that pairs the electrons can no longer be phonons and still remains unknown. Actually, it can be proved that any attractive force between the electrons, as small it could be, would produce their Cooper pairing [36].

Therefore the problem of HTS can be traced back to that of identifying the force that links the electrons. We suggest that this force actually derives from a quantum potential.

Most HTS are copper oxide compounds in which superconductivity arises when they are doped either by extra charges but more often by ‘holes’ (positive charge carrier). Moreover, a systematic electronic inhomogeneity has been reported at the microscopic level, in particular in compounds like Bi_2_Sr_2_CaCu_2_O_8+_*_x_* [37], the local density of states (LDOS) showing ‘hills’ and ‘valley’ of size ∼30 Angstroms, strongly correlated with the SC gap. Actually, the minima of LDOS modulations preferentially occur at the dopant defects [38]. The regions with sharp coherence peaks, usually associated with strong superconductivity, are found to occur between the dopant defect clusters, near which the SC coherence peaks are suppressed.

Basing ourselves on these observations, we have suggested that, at least in this type of compound, the electrons can be trapped in the quantum potential well created by these electronic modulations.

Let us give here a summary of this new proposal. We denote by *ψ_n_* the wave function of doping charges which have diffused from the initial site of dopant defects, and by *ψ_s_* the wave function of the fraction of carriers which will be tied in Cooper pairs (only 19%–23% of the total doping induced charge joins the superfluid near optimum doping).

We set *ψ_n_* = *ψ_s_* + *ψ_d_*, where *ψ_d_* is the wave function of the fraction of charges which do not participate in the superconductivity.

The doping induced charges constitutes a quantum fluid which is expected to be the solution of a Schrödinger equation (here of standard QM, *i.e.*, written in terms of the microscopic Planck's constant ℏ)

(51) $$\frac{\hbar^{2}}{2m}\Delta \psi_{n}+i\hbar \frac{\partial \psi_{n}}{\partial t}=\phi \psi_{n}$$

where *ϕ* is a possible external scalar potential, and where we have neglected the magnetic effects as a first step.

Let us separate the two contributions *ψ_s_* and *ψ_d_* in this equation. We obtain:

(52) $$\frac{\hbar^{2}}{2m}\Delta \psi_{s}+i\hbar \frac{\partial \psi_{s}}{\partial t}-\phi \;\psi_{s}=-\frac{\hbar^{2}}{2m}\Delta \psi_{d}-i\hbar \frac{\partial \psi_{d}}{\partial t}+\phi \;\psi_{d}$$

We can now introduce explicitly the probability densities *n* and the phases *θ* of the wave functions $\psi_{s}=\sqrt{n_{s}}\times e^{i\theta_{s}}$ and $\psi_{d}=\sqrt{n_{d}}\times e^{i\theta_{d}}$. The velocity fields of the (s) and (d) quantum fluids are given by *V_s_* = (ℏ/*m*)∇*θ_s_* and *V_d_* = (ℏ/*m*)∇*θ_d_*. As we have seen above, a Schrödinger equation can be put into the form of fluid mechanics-like equations, its imaginary part becoming a continuity equation and the derivative of its real part becoming a Euler equation with quantum potential. Therefore the above equation can be written as:

(53) $$\frac{\partial V_{s}}{\partial t}+V_{s}.\nabla V_{s}=-\frac{\nabla \phi }{m}-\frac{\nabla Q_{s}}{m}-\left(\frac{\partial V_{d}}{\partial t}+V_{d}.\nabla V_{d}+\frac{\nabla Q_{d}}{m}\right)$$

(54) $$\frac{\partial n_{s}}{\partial t}+\text{div}(n_{s}V_{s})=-\frac{\partial n_{d}}{\partial t}-\text{div}(n_{d}V_{d})$$

But the (d) part of the quantum fluid, which is not involved in the superconductivity, remains essentially static, so that *V_d_* = 0 and *∂n_d_*/*∂t* = 0. Therefore we obtain for the quantum fluid (s) a new system of fluid equations:

(55) $$\frac{\partial V_{s}}{\partial t}+V_{s}\text{.}\nabla V_{s}=-\frac{\nabla \phi }{m}-\frac{\nabla Q_{s}}{m}-\frac{\nabla Q_{d}}{m}$$

(56) $$\frac{\partial n_{s}}{\partial t}+\text{div}(n_{s}V_{s})=0$$

which can be re-integrated under the form of a Schrödinger equation

(57) $$\frac{\hbar^{2}}{2m}\Delta \psi_{s}+i\hbar \frac{\partial \psi_{s}}{\partial t}-(\phi +Q_{d})\psi_{s}=0$$

It therefore describes the motion of electrons (s), represented by their wave function *ψ_s_*, in a potential well given by the exterior potential *ϕ*, but also by an interior quantum potential *Q_d_* which just depends on the local fluctuations of the density *n_d_* of charges,

(58) $$Q_{d}=-\frac{\hbar^{2}}{2m}\frac{\Delta \sqrt{n_{d}}}{\sqrt{n_{d}}}$$

Even if in its details this rough model is probably incomplete, we hope this proposal, according to which the quantum potential created by the dopants provides the attractive force needed to link electrons into Cooper pairs, to be globally correct, at least for some of the existing HT superconductors.

Many (up to now) poorly understood features of cuprate HTS can be explained by this model. For example, the quantum potential well involves bound states in which two electrons can be trapped with zero total spin and momentum. One can show that the optimal configuration for obtaining bound states is with 4 dopant defects (oxygen atoms), which bring 8 additional charges. One therefore expects a ratio *n_s_*/*n_n_* = 2/(8 + 2) = 0.2 at optimal doping. This is precisely the observed value [39], for which, to our knowledge, no explanation existed up to now.

The characteristic size of LDOS wells of ∼30 Angstroms is also easily recovered in this context: the optimal doping being *p* = 0.155 = 1/6.5, the 8 to 10 charges present in the potential well correspond to a surface (8 − 10) × 6.5 = (52 − 65) = (7.2 − 8.1)^2^ in units of *d*_CuO_ = 3.9 Angstroms, *i.e.*, 28–32 Angstroms as observed experimentally.

In this context, the high critical temperature superconductivity would be a geometric multiscale effect. In normal SC, the various elements which permit the superconductivity, Cooper pairing of electrons, formation of a quantum bosonic fluid and coherence of this fluid are simultaneous. In HTS, under the quantum potential hypothesis, these elements would be partly disconnected and related to different structures at different scales (in relation to the connectivity of the potential wells), achieving a multi-scale fractal structure [40].

If confirmed, this would be a nice application of the concept of quantum potentials [41], here in the context of standard microscopic quantum mechanics.

## 5. Scale Relativity in Non-differentiable Velocity-Space

### 5.1. Analogy between Turbulence and Living Systems

Living systems are well known to exhibit fractal structures from very small scales up to the organism size and even to the size of the collective entities (e.g., a forest made of trees). Therefore it is relevant to assess and quantify these properties with sophisticated models.

Some advanced fractal and multifractal models have been developed in the field of turbulence because fractals are the basic fundamental feature of chaotic fluid dynamics [42]. They have been described since the famous law of Kolmogorov, known as K41 [43]. In the atmosphere, scale laws are observed from micrometers up to thousands of kilometers. Turbulence can be described as flow of energy injected at large scale that cascades into smaller and smaller structures. This process redirects the energy into all directions and it is ultimately dissipated into heat at the smallest scale.

There is therefore a strong analogy with living systems. An investigation of turbulence *versus* living systems is particularly interesting as there are a number of common points:

- Dissipation: both turbulent flows and living systems are dissipative.
- Non-isolated: existence of source and sink of energy.
- Out of equilibrium.
- Chaotic.
- Existence of stationary structures. Individual “particles” enter and go out in a very complex way, while the overall structure grows (growth of living systems, development of turbulence) then remains stable on a long time scale.
- Fundamentally multi-scale and multi-fractal structuring.
- Injection of energy at an extreme scale with dissipation at the other (the direction of the multiplicative cascade is reversed in living systems compared to laboratory turbulence). *etc*.

### 5.2. Application of Scale Relativity to Turbulence

In a recent work, L. de Montera has suggested an original application of the scale relativity theory to the yet unsolved problem of turbulence in fluid mechanics [44]. He has remarked that the Kolmogorov scaling of velocity increments in a Lagrangian description (where one follows an element of fluid, for example thanks to a seeded micro particle [45]),

(59) $$\delta \upsilon \propto {\left|\delta t\right|}^{1/2}$$

was exactly similar to the fractal fluctuation Equation (8) which is at the basis of the scale relativity description.

The difference is that coordinates remain differentiable, while in this new context velocity becomes non-differentiable, so that accelerations *a* = *δυ*/*δt* ∝ |*δt*|^−1/2^ become scale-divergent. Although this power law divergence is clearly limited by the dissipative Kolmogorov small scale, it is nevertheless fairly supported by experimental data, since acceleration of up to 1500 times the acceleration of gravity have been measured in turbulent flows [45,46]).

De Montera's suggestion therefore amounts to apply the scale relativity method after an additional order of differentiation of the equations. The need for such a shift has already been remarked in the framework of stochastic models of turbulence [47,48].

Let us consider here some possible implications of this new, very interesting, proposal.

The necessary conditions which underlie the construction of the scale relativity covariant derivative are very clearly fulfilled for turbulence (now in velocity space):

(1) The chaotic motion of fluid particles implies an infinity of possible paths.
(2) Each of the paths (realizations of which are achieved by test particles of size <100 μm in a Lagrangian approach, [46]) are of fractal dimension *D_F_* = 2 in velocity space, at least in the K41 regime (Equation (59)).
(3) The two-valuedness of acceleration is manifested in turbulence data. As remarked by Falkovich *et al.* [49], the usual statistical tools of description of turbulence (correlation function, second order structure function, *etc.*) are reversible, while turbulence, being a dissipative process, is fundamentally irreversible. The two-valuedness of derivative is just a way to account for the symmetry breaking under the time scale reflexion *δt* → −*δt*. Among the various ways to describe this doubling [50], one of them is particularly adapted to comparison with turbulence data. It consists of remarking that the calculation of a derivative involves a Taylor expansion
  (60) $$\frac{dX}{dt}=\frac{X(t+dt)-X(t)}{dt}=\frac{\left(X(t)+X^{\prime }(t)dt+\frac{1}{2}X^{\prime \prime }(t)dt^{2}+\ldots )-X(t\right)}{dt}$$
  so that one obtains
  (61) $$\frac{dX}{dt}=X^{\prime }(t)+\frac{1}{2}X^{\prime \prime }(t)dt+\ldots$$
  For a standard non-fractal function, the contribution $\frac{1}{2}X^{\prime \prime }(t)dt$ and all the following terms of higher order vanish when *dt* → 0, so that one recovers the usual result *dX*/*dt* = *X′*(*t*). But for a fractal function such that its second derivative is scale divergent as *X″*(*t*) ∝ 1/*dt*, the second order term can no longer be neglected and must contribute to the definition of the derivative ([8], Section 3.1).Therefore one may write
  (62) $$\begin{array}{ll} \frac{d_{+}X}{dt}=X^{\prime }(t)+\frac{1}{2}X^{\prime \prime }(t)|dt|, & \frac{d_{-}X}{dt}=X^{\prime }(t)-\frac{1}{2}X^{\prime \prime }(t)|dt| \end{array}$$
  then
  (63) $$\frac{\hat{d}X}{dt}=\frac{d_{+}+d_{-}}{2dt}X-i\frac{d_{+}-d_{-}}{2dt}X=X^{\prime }(t)-i\frac{1}{2}X^{\prime \prime }(t)|dt|$$
  Lagrangian measurements of turbulence data [51,52] confirm this expectation. One finds that the acceleration *a* = *υ′* and its increments *da* = *υ″dt* are indeed of the same numerical order: in these data, the dispersions are respectively *σ_a_* = 280 m/s^2^ vs *σ_da_* = 220 m/s^2^. This fundamental result fully supports the acceleration two-valuedness on an experimental basis.
(4) The dynamics is Newtonian: the equation of dynamics in velocity space is the time derivative of the Navier-Stokes equation, *i.e.*,
  (64) $$\frac{da}{dt}=\dot{F}$$
  Langevin-type friction terms may occur in this equation but they do not change the nature of the dynamics. They will simply add a non-linear contribution in the final Schrödinger equation.
(5) The range of scales is large enough for a K41 regime to be established: in von Karman laboratory fully developed turbulence experiments, the ratio between the small dissipative scale and the large (energy injection) scale is larger than 1000 and a K41 regime is actually observed [51].

The application of the scale relativity method is therefore fully supported experimentally in this case. Velocity increments *dV* can be decomposed into two terms, a classical differentiable one *dυ* and a fractal fluctuation:

(65) $$dV=d\upsilon +\zeta \sqrt{2D_{\upsilon }\;dt}$$

where < *ζ* > = 0 and < ζ^2^ >= 1. One recognizes here the K41 scaling in *dt*^1/2^. One introduces a complex acceleration field 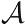 = *a* − *i* (*da*/2) and a total ‘covariant’ derivative

(66) $$\frac{\hat{d}}{dt}=\frac{\partial }{dt}+\text{A}.\nabla_{\upsilon }-i{\text{D}}_{\upsilon }\Delta_{\upsilon }$$

and then write a super-dynamics equation

(67) $$\frac{\hat{d}}{dt}\text{A}=\dot{F}$$

A wave function *ψ* acting in velocity space can be constructed from the acceleration field,

(68) $$\text{A}=-2i{\text{D}}_{\upsilon }\nabla_{\upsilon }\ln\psi$$

and the super-dynamics equation can then be integrated under the form of a Schrödinger equation including possible non-linear terms (NLT)

(69) $${\text{D}}_{\upsilon }^{2}\;\Delta_{\upsilon }\psi +i{\text{D}}_{\upsilon }\frac{\partial \psi }{\partial t}=\frac{\phi }{2}\psi +\text{NLT}$$

where *ϕ* is a potential (in velocity space) from which the force *Ḟ* or part of this force derives.

By coming back to a fluid representation—but now in terms of the fluid of potential paths—using as variables *P*(*υ*) = l*ψ*l^2^ and *a*(*υ*) (which derives from the phase of the wave function), this equation becomes equivalent to the combination of a Navier-Stokes-like equation written in velocity space and a continuity equation,

(70) $$\frac{da}{dt}=\dot{F}+2{\text{D}}_{\upsilon }^{2}\nabla_{\upsilon }\left(\frac{\Delta_{\upsilon }\sqrt{P}}{\sqrt{P}}\right)$$

(71) $$\frac{\partial P}{\partial t}+{\text{div}}_{\upsilon }\;(Pa)=0$$

Therefore we have recovered the same equation from which we started (time derivative of Navier-Stokes equation) but a new term has emerged, namely, a quantum-type force which is the gradient of a quantum-type potential in velocity space. One can now re-integrate this equation, and one thus obtains the initial Navier-Stokes equation (in the uncompressible case *ρ* = 1 and with a viscosity coefficient *ν*):

(72) $$\left(\frac{\partial }{\partial t}+\upsilon \cdot \nabla \right)\;\upsilon =-\nabla p+\nu \Delta \upsilon +2{\text{D}}_{\upsilon }^{2}\int_{0}^{t}\nabla_{\upsilon }\left(\frac{\Delta \upsilon \sqrt{P}}{\sqrt{P}}\right)\;dt$$

but with an additional term which manifests the fractality of the flow in velocity space. The value of 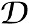
*_υ_* is directly given, in the K41 regime, by the parameter which commands the whole process, the energy dissipation rate by unit of mass, *ε*,

(73) $$2{\text{D}}_{\upsilon }=C_{0}\varepsilon$$

where *C*_0_ is Kolmogorov's numerical constant (whose estimations vary from 4 to 9). Concerning the two small scale (dissipative) and large scale (energy injection) transitions, one could include them in a scale varying 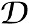
*_υ_*, but a better solution consists of keeping 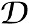
*_υ_* constant, then to include the transitions subsequently to the whole process in a global way.

The intervention of such a missing term in developed turbulence is quite possible and is even supported by experimental data. Indeed, precise experimental measurements of one of the numerical constants which characterize the universal scaling of turbulent flows, $a_{0}=\nu^{1/2}\varepsilon^{-3/2}\sigma_{a}^{2}$, has given constant values around *a*_0_ = 6 in the developed turbulence domain *R*_λ_ ≥ 500 [46]. However, in the same time, direct numerical simulations (DNS) of Navier-Stokes equations under the same conditions [53,54,55] have systematically given values around *a*_0_ = 4, smaller by a factor 2/3.

Let us derive the scale relativity prediction of this constant. We indeed expect an additional contribution to the DNS, since they use the standard NS equations and do not include the new quantum potential.

The considered experiments are van Karman-type flows. The turbulence is generated in a flow of water between counter-rotating disks (with the same opposite rotational velocity) in a cylindrical container [46]. For such experiments the Lagrangian velocity distribution is given with a good approximation by a Gaussian distribution [51,52] centered on *υ* = 0 (in the laboratory reference system). We can therefore easily calculate the velocity quantum potential. We find for these specific experiments

(74) $$Q_{\upsilon }=-{\text{D}}_{\upsilon }^{2}\;\left(\frac{\upsilon^{2}-6\sigma_{\upsilon }^{2}}{2\sigma_{\upsilon }^{4}}\right)$$

where $\sigma_{\upsilon }^{2}$ is the velocity variance. Therefore the quantum-like force reads ${\text{F}}_{Q\upsilon }=-\nabla_{\upsilon }Q_{\upsilon }=({\text{D}}_{\upsilon }^{2}/\sigma_{\upsilon }^{4})\text{v}$, and the additional term in Navier-Stokes equations finally reads, in a reference system whose origin is the center of the cylinder,

(75) $${\text{F}}_{Qx}=\int_{0}^{t}{\text{F}}_{Q\upsilon }dt=\frac{{\text{D}}_{\upsilon }^{2}}{\sigma_{\upsilon }^{4}}\text{x}$$

which is just a repulsive harmonic oscillator force. We therefore expect a new geometric contribution to the acceleration variance:

(76) $$\sigma_{a}^{2}={\left(\sigma_{a}\right)}_{cl}^{2}+\frac{{\text{D}}_{\upsilon }^{4}}{\sigma_{\upsilon }^{8}}\sigma_{x}^{2}$$

Now the parameter 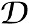
*_υ_* = *C*_0_*ε*/2 can also, in the K41 regime, be written in function of *σ_υ_* and of the Lagrangian integral time scale *T_L_* as ${\text{D}}_{\upsilon }=\sigma_{\upsilon }^{2}/T_{L}$, while we can take *σ_x_* ≈ *L*, the Lagrangian length scale, and we obtain the simple expression

(77) $$\frac{{\left(\sigma_{a}\right)}_{cl}^{2}}{\sigma_{a}^{2}}=1-\frac{L^{2}}{\sigma_{a}^{2}T_{L}^{4}}.$$

This ratio (l.h.s. of this relation) has been observed to be ≈2/3 by Voth *et al.* [46] (taking the DNS values for (σ*_a_*)*_cl_* and the experimental ones for *σ_a_*). The experimental values of *L*, *σ_a_* and *T_L_* (fitted from the published data) for the same experiments [46] yield values of the r.h.s. that are also around 2/3, a very satisfactory agreement between the theoretical expectation and the experimental result.

For example, in one of the experiments with *R*_λ_ = 690, Voth *et al.* have measured *σ_a_* = 87 m/s^2^ and *L* = 0.071 m [46], while the fitted Lagrangian time scale is found to be *T_L_* = 39 ms, so that $L/(\sigma_{a}T_{L}^{2})=0.54$ and its square is ≈ 1/3. For the same experiment, (*a*_0_)_DNS_ = 4.5 while (*a*_0_)_exp_ = 6.2, so that (1 − (*a*_0_)_DNS_/(*a*_0_)_exp_)^1/2^ = 0.52, very close to the theoretical expectation from the scale relativity correction (0.54).

Although this is not yet a definitive proof of a quantum-like regime in velocity space for developed turbulence (which we shall search in a finer analysis of turbulence data), this adequation is nevertheless a very encouraging result in favor of de Montera's proposal [44].

## 6. Applications

Let us now give some explicit examples of applications of the scale relativity theory in life sciences, with special emphasis to cases where the cell scale is directly or indirectly concerned.

Actually this theory, through its generalized scale laws and its motions laws that take the form of macroscopic quantum-type laws, allows one to naturally obtain from purely theoretical arguments some functions, characteristics and fundamental processes which are generally considered as specific of living systems. We shall briefly consider the following ones (in a non-exhaustive way): confinement, morphogenesis, spontaneous organization, link to the environment, “quantization”, duplication, branching, (log-periodic) evolution, multi-scale integration (see [1,2,8,14,15] for more details).

### 6.1. Quantization of Structures

Living systems are often characterized by properties of “quantization” and discretization at a very fundamental level. We mean here that they are organized in terms of specific structures having characteristic sizes that are defined in a limited range of scales. The example of cells, which can be considered a kind of “biological quantum”, is the most clear, but this is also true of the cell nucleus, of organs and of organisms themselves for a given species.

This kind of property is naturally expected from the scale relativity approach. Indeed,, the three conditions under which the fundamental equation of dynamics is transformed in a Schroödinger equation (infinity or very large number of potential paths, fractality of these paths and infinitesimal irreversibility) could reasonably be achieved, at least as approximations, in many biological systems.

**Figure 1:**
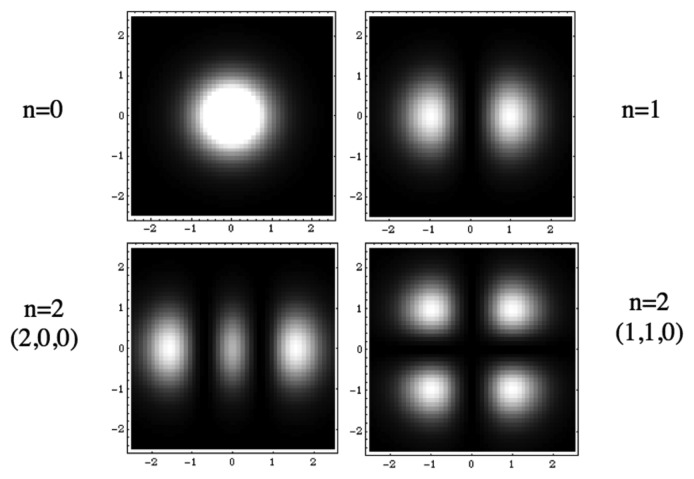
Example of quantized structures: solutions of a Schrödinger equation for an harmonic oscillator potential.

Such a Schrödinger equation yields stationary and stable solutiöns only for some discretized values of the parameters (energy, momentum, angular momentum, *etc.*). This remains true of the macroscopic one obtained in scale relativity. These quantized solutions, yielding probability density functions, are solutions of the time-independent equation, written for these particular values of the parameters, in particular of the energy. Now these probability densities define characteristic structures, for example in terms of peaks of probability (see Figure 1). Therefore this property can be viewed as a natural tendency for such a system to structure, and this in a “quantized” way [10].

These structures and their type of quantization appear in dependence of the various limit conditions (in time and space) and environment conditions (presence of forces and fields). This is a very appealing result for biology, since it is clear that not all possible shapes are achieved in nature, but only those corresponding to definite organization bauplans, and that these bauplans appear in relation with the environmental conditions. This is manifest in particular in terms of species punctuated selection and evolution. Some examples will be given in what follows.

### 6.2. Confinement and Cell Wall

As we have seen in the theoretical part of this paper (Section 2.1), one can obtain usual fractal or multifractal scale laws as solutions of first order differential equations acting in the scale space. But these laws can also be generalized to a “scale dynamics” involving second order differential equations. As we have already remarked, this is similar to the passage from inertial laws to Newton's laws of dynamics as concerns motion. Pushing further the analogy, the deviation from a constant fractal dimension (corresponding to scale invariance) can be attributed to the action of a “scale force”.

A particularly interesting application to biology is the case when this force is given by an harmonic oscillator. Indeed, harmonic oscillators appear in a very common way, since they describe the way a system evolves after having been removed from its equilibrium position. But here, the “position” is a scale, which means that, in the case of an attractive oscillator, the system will change its scale in a periodic way. This may yield model of breath/lung dilation and contraction. An interesting feature of such models is that the scale variable is logarihmic, so that the dilation/contraction remains symmetrical only for small deviations from equilibrium, while it becomes disymmetrical for larger ones, as observed in actual situations.

**Figure 2:**
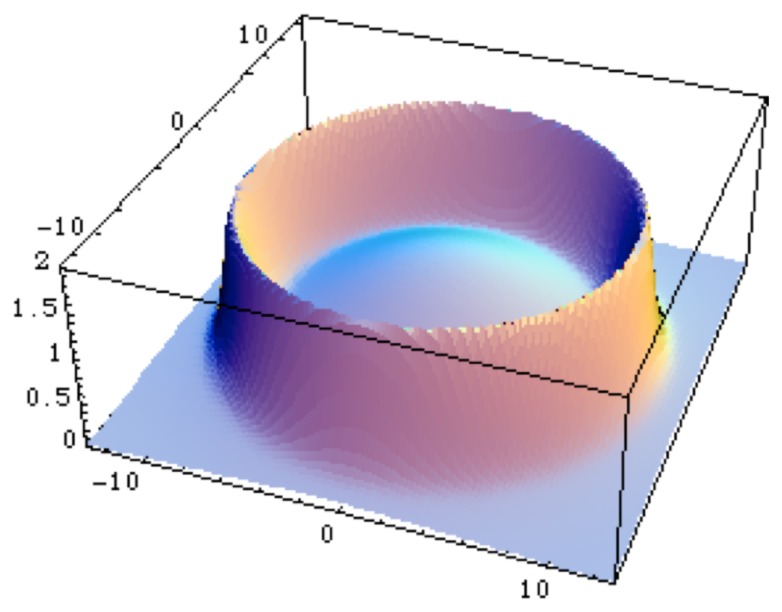
A model of cell wall. The figure gives the value of the fractal dimension which is solution of a second order scale differential equation involving a repulsive harmonic oscillator in scale space. One finds a constant fractal dimension in the inner region, a diverging dimension in an intermediate region which may represent a “wall”, then another constant dimension (possibly non-fractal) in the outer region.

In the case of a repulsive oscillator, one obtains a three-domain system, characterized by a inner and an outer fractal dimension which may be different, separated by a zone at intermediate scales where the fractal dimension diverges (see Figure 2). When one identifies the scale variable as a distance to a center, it describes a system in which has emerged a clear separation between an inner and an outer region, which is one of the properties of the first prokaryotic cell.

Moreover, the zone where the fractal dimension rapidly increases (up to divergence in the mathematical model) corresponds to an increased ‘thickness’ of the material and it can therefore be interpreted as the description of a ‘membrane’. It is indeed the very nature of biological systems to have not only a well-defined size and a well-defined separation between interior and exterior, but also systematically an interface between them, such as membranes or walls. This is already true of the simplest prokaryote living cells. Therefore this result suggests that there could be a connection between the existence of a scale field (for example a pressure), the confinement of the cellular material and the appearance of a limiting membrane or wall [1,8].

This is reminiscent of eukaryotic cellular division which involves both a dissolution of the nucleus membrane and a deconfinement of the nucleus material, transforming, before the division, an eukaryote into a prokaryote-like cell. This could be a key toward a better understanding of the first major evolutionary leap after the appearance of cells, namely the emergence of eukaryotes.

### 6.3. Morphogenesis

The Schrödinger equation, which is the form taken by the equation of dynamics after integration in scale relativity, can be viewed as a fundamental equation of morphogenesis. It has not been yet considered as such, because its unique domain of application was, up to now, the microscopic domain concerned with molecules, atoms and elementary particles, in which the available information was mainly about energy and momentum.

However, scale relativity extends the potential domain of application of Schrödinger-like equations to every systems in which the three conditions (1) infinite or very large number of trajectories; (2) fractal dimension of individual trajectories; (3) local irreversibility, are fulfilled. Macroscopic Schrödinger equations can be constructed, which are not based on Planck's constant ℏ, but on constants that are specific of each system (and may emerge from their self-organization). In addition, systems which can be described by hydrodynamics equations including a quantum-like potential also come under the generalized macroscopic Schrödinger approach.

The three above conditions seem to be particularly well adapted to the description of living systems. Let us give a simple example of such an application.

In living systems, morphologies are acquired through growth processes. One can attempt to describe such a growth in terms of an infinite family of virtual, fractal and locally irreversible, fluid-like trajectories. Their equation can therefore be written under the form of a fractal geodesic equation, then it can be integrated as a Schrödinger equation or, equivalently, in terms of hydrodynamics-type energy and continuity equations including a quantum-like potential. This last description therefore shares some common points with recent very encouraging works in embryogenesis which describe the embryo growth by visco-elastic fluid mechanics equations [56,57]. The addition of a quantum potential to these equations would give them a Schrödinger form, and therefore would allow the emergence of quantized solutions. This could be an interesting advantage for taking into account the organization of living systems in terms of well defined bauplans [58] and the punctuated evolution of species whose evolutive leaps go from one organization plan to another [59].

Let us take a more detailed example of morphogenesis. If one looks for solutions describing a growth from a center, one finds that this problem is formally identical to the problem of the formation of planetary nebulae, and, from the quantum point of view, to the problem of particle scattering, e.g., on an atom. The solutions correspond to the case of the outgoing spherical probability wave.

Depending on the potential, on the boundary conditions and on the symmetry conditions, a large family of solutions can be obtained. Considering here only the simplest ones, *i.e.*, central potential and spherical symmetry, the probability density distribution of the various possible values of the angles are given in this case by the spherical harmonics,

(78) $$P(\theta ,\varphi )={\left|Y_{lm}(\theta ,\varphi )\right|}^{2}$$

These functions show peaks of probability for some quantized angles, depending on the quantized values of the square of angular momentum *L*^2^ (measured by the quantum number *l*) and of its projection *L_z_* on axis *z* (measured by the quantum number *m*).

**Figure 3:**
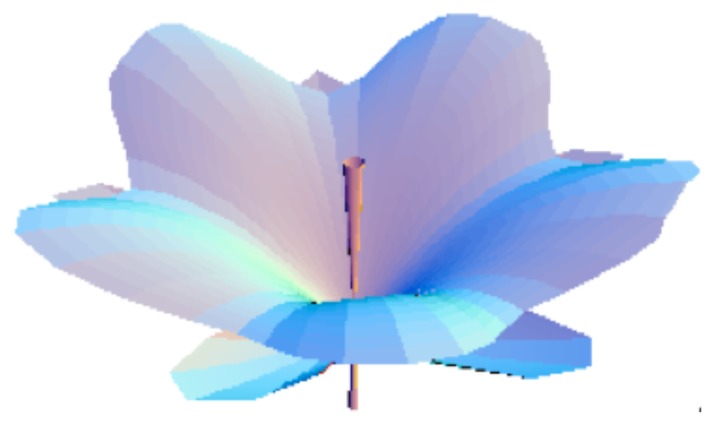
Morphogenesis of a ‘flower’-like structure, solution of a Schrödinger equation that describes a growth process from a center (*l* = 5, *m* = 0). The ‘petals’, ‘sepals’ and ‘stamen’ are traced along angles of maximal probability density. A constant force of ‘tension’ has been added, involving an additional curvature of ‘petals’, and a quantization of the angle *θ* that gives an integer number of ‘petals’ (here, *k* = 5).

**Figure 4:**
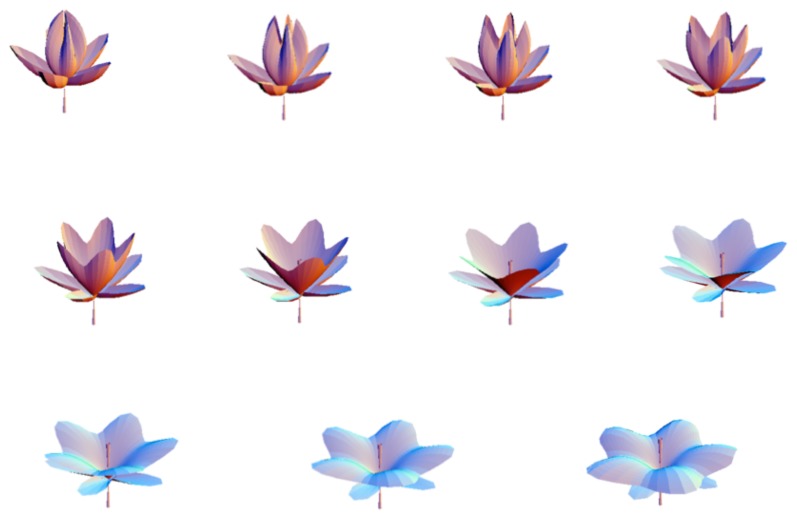
Steps in the “opening” of the flower-like structure of Figure 3. The various shapes are all solutions of a Schrödinger equation derived from the scale relativity equation of dynamics written for a growth process coming from a center. The opening here is just a result of the balance between the action of gravity and the inner force of tension.

Finally the ‘most probable’ morphology is obtained by ‘sending’ matter along angles of maximal probability. The biological constraints leads one to skip to cylindrical symmetry. This yields in the simplest case a periodic quantization of the angle *θ* (measured by an additional quantum number *k*), that gives rise to a separation of discretized ‘petals’. Moreover there is a discrete symmetry breaking along the *z* axis linked to orientation (separation of ‘up’ and ‘down’ due to gravity, growth from a stem). The solutions obtained in this way show floral ‘tulip’-like shapes (see Figure 3 and Figure 4 and [2,15,24]).

### 6.4. Duplication

Another very interesting feature of quantum-type systems (in the present context of their possible application to biology) is their behavior under a change of energy. Indeed, while the fundamental level solution of a stationary Schrödinger equation describes a single structure, the first excited solution is usually double.

**Figure 5:**
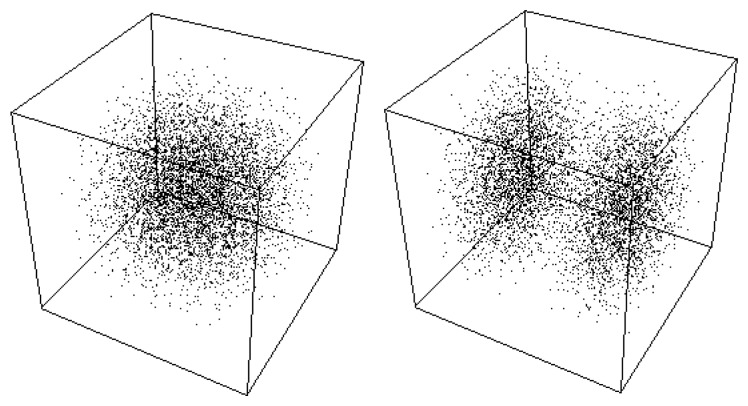
Model of duplication. The stationary solutions of the Schrödinger equation in a 3D box can take only discretized morphologies in correspondence with quantized values of the energy. An increase of energy results in a jump from a single structure to a binary structure. No stable solution can exist between the two structures.

**Figure 6:**
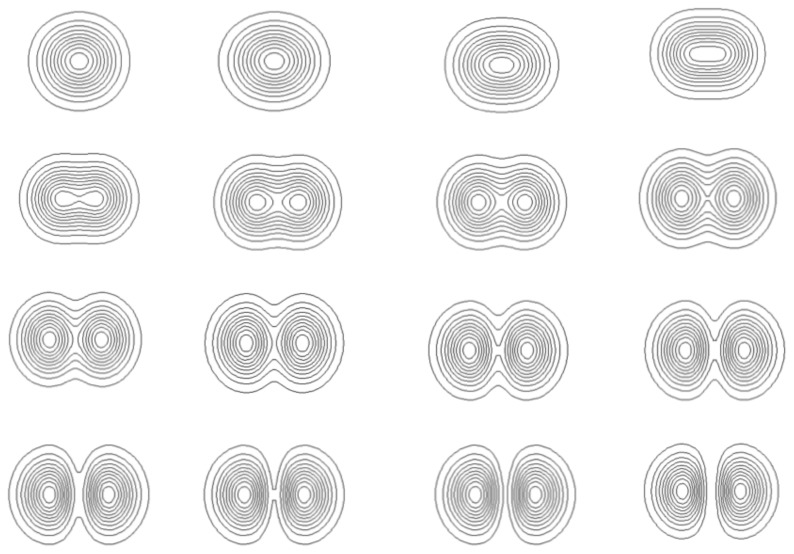
Steps of duplication. Stationary solutions of the Schrödinger equation can take only discretized morphologies in correspondence with quantized values of the energy. The successive figures (from top left to bottom right) give different steps of the division process, obtained as solutions of the time-dependent Schödinger equation in an harmonic oscillator potential, which jump from the fundamental level (top left) to the first excited level (bottom right). These extreme solutions are stable (stationary solution of the time-independent Schrödinger equation), while the intermediate solutions are transitory. Therefore it is seen that the system spontaneously jumps from the one structure to the two-structure morphology.

**Figure 7:**
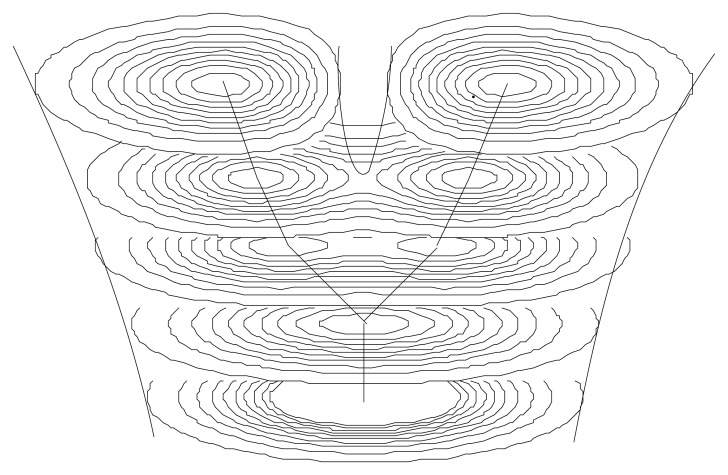
Model of branching and bifurcation. Successive solutions of the time-dependent 2D Schrödinger equation in an harmonic oscillator potential are plotted as isodensities. The energy varies from the fundamental level (*n* = 0) to the first excited level (*n* = 1), and, as a consequence, the system jumps from a one-structure to a two-structure morphology.

Therefore, the passage from the fundamental (‘vacuum’) level to the first excited level provides us with a (rough) model of duplication/cellular division (see Figure 5, Figure 6 and Figure 7). The quantization of the solutions implies that, in case of energy increase, the system will not increase its size, but will instead be lead to jump from a single structure to a binary structure, with no stable intermediate step between the two stationary solutions *n* = 0 and *n* = 1, since the energy of the stationary solutions is itself quantized. Moreover, if one comes back to the scale relativity level of description of individual paths (whose velocity field constitutes the wave function while the equation of dynamics becomes a Schr'ödinger equation), one finds that from each point of the initial one body-structure there exist trajectories that go to the two final structures. In this framework, duplication is expected to be linked to a discretized and precisely fixed jump in energy.

It is clear that, at this stage, such a model is extremely far from describing the complexity of a true cellular division, which it did not intend to do. Its interest is to be a generic and general model for a spontaneous duplication process of quantized structures, linked to energy jumps. Indeed, the jump from one to two probability peaks when going from the fundamental level to the first excited level is found in many different situations of which the harmonic oscillator and the 3D box cases are only examples. Moreover, this property of spontaneous duplication is expected to be conserved under more elaborated versions of the description provided the asymptotic small scale behavior remains of constant fractal dimension *D_F_* ≈ 2, such as, e.g., in cell wall-like models based on a locally increasing effective fractal dimension.

### 6.5. Bifurcation, Branching Process

Such a model can also be applied to a first rough description of a branching process (Figure 7), e.g., in the case of a tree growth when the previous structure remains instead of disappearing as in cell duplication.

Such a model is still clearly too rough to claim that it truly describes biological systems. It is just intended to describe a general, spontaneous functionality. But note that it may be improved and complexified by combining with it and integrating various other functions and processes generated by the scale relativity approach. For example, one may apply the duplication or branching process to a system whose underlying scale laws include (i) a model of membrane—or cell wall—through a fractal dimension that becomes variable with the distance to a center; (ii) a model of multiple hierarchical levels of organization depending on ‘complexergy’ (see below).

### 6.6. Origin of Life: A New Approach

A fundamentally new feature of the scale relativity approach as concerns the question of the origin of life is that the Schrödinger form taken by the geodesic equation can be interpreted as a general tendency for systems to which it applies to make structures, *i.e.*, to naturally lead to self-organization and neguentropy. In the framework of a classical deterministic approach, the question of the formation of a system is always posed in terms of initial conditions. In the new framework, the general existence of stationary solutions allows structures to be formed whatever the initial conditions, in correspondence with the field, the symmetries and the boundary conditions (which become the environmental conditions in biology), and in function of the values of the various conservative quantities that characterize the system.

Such an approach could allow one to ask the question of the origin of life in a renewed way. The emergence of life may be seen as an analog of the ‘vacuum’ (lowest energy) solutions in a quantum-type description, *i.e.*, of the passage from a non-structured medium to the simplest, fundamental level structures. In astrophysics and cosmology, the problem amounts to understand the apparition, from the action of gravitation alone, of structures from a highly homogeneous and non-structured medium. In the standard approach to this problem a large quantity of postulated and unobserved dark matter is needed to form structures, and even with this help the result is dissatisfying. In the scale relativity framework, we have suggested that an underlying fractal geometry of space involves a Schrödinger form for the equation of motion, leading both to a natural tendency to form structures and to the emergence of an additional potential energy which may explain the effects usually attributed to a missing mass [8,10].

The problem of the origin of life, although clearly far more difficult and complex, shows common features with this question of structure formation in cosmology. In both cases one needs to understand the apparition of new structures, functions, properties, *etc*… from a medium which does not yet show such structures and functions. In other words, one need a theory of emergence. We hope that scale relativity is a good candidate for such a theory, since it owns the two required properties: (i) for problems of origin, it gives the conditions under which a weakly struct ng or destructuring (e.g., diffusive) classical system may become quantum-like and therefore structuring; (ii) for problems of evolution, it makes use of the spontaneous self-organizing property of the quantum-like theory.

**Figure 8:**
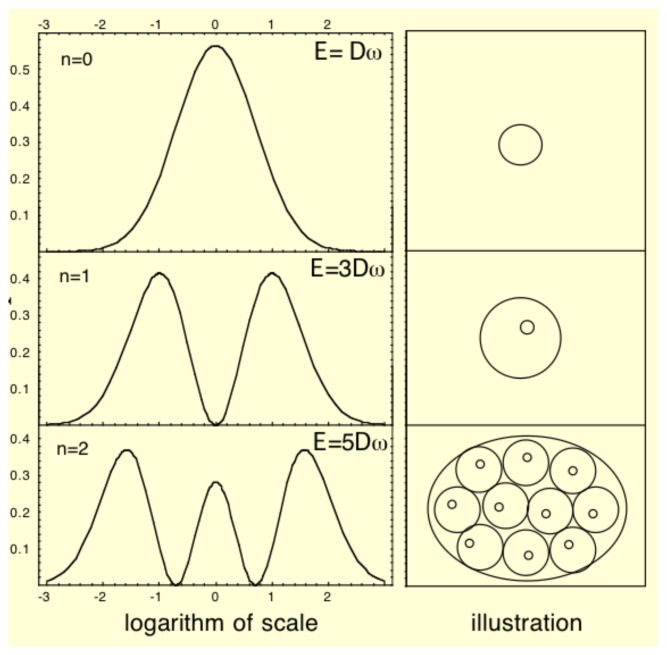
Schematic illustration of a model of hierarchical organization based on a Schrödinger equation acting in scale space. The fundamental mode corresponds to only one level of hierarchy, while the first and second excited modes describe respectively two, then three embedded hierarchical structures.

We have therefore tentatively suggested a new way to tackle the question of the origin of life (and in parallel, of the present functioning of the intracellular medium) [8,60]. The prebiotic medium on the primordial Earth is expected to have become chaotic. As a consequence, on time scales long with respect to the chaos time (horizon of predictability), the conditions which underlie the transformation of the motion equation into a Schrödinger-type equation become fulfilled (complete information loss on angles, position and time leading to a fractal dimension 2 behavior on a range of scales reaching a ratio of at least 10^4^−10^5^, see ([8], Chapter 10). Since the chemical structures of the prebiotic medium have their lowest scales at the atomic size, this means that, under such a scenario, one expects the first organized units to have appeared at a scale of about 10 μm, which is indeed a typical scale for the first observed prokaryotic cells (see Figure 8).

The spontaneous transformation of a classical, possibly diffusive mechanics, into a quantum-like mechanics, with the diffusion coefficient becoming the quantum self-organization parameter 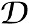 would have immediate dramatic consequences: quantization of energy and energy exchanges and therefore of information, apparition of shapes and quantization of these shapes (the cells can be considered as the ‘quanta’ of life), spontaneous duplication and branching properties (see following sections), *etc*… Moreover, due to the existence of a vacuum energy in a quantum-type mechanics (*i.e.*, of a non-vanishing minimal energy for a given system), we expect the primordial structures to appear at a given non-zero energy, without any intermediate step.

In such a framework, the fundamental equation would be the equation of molecular fractal geodesics, which could be transformed into a Schrödinger equation for wave functions *ψ*. This equation describes a universal tendency to make structures in terms of a probability density *P* for chemical products (constructed from the distribution of geodesics), given by the squared modulus of the wave function $\psi =\sqrt{P}\times e^{i\theta }$. Each of the molecules being subjected to this probability (which therefore plays the role of a potentiality), it is proportional to the concentration *c* for a large number of molecules, *P* ∝ *c*.

Finally, the Schrödinger equation may in its turn be transformed into a continuity and Euler hydrodynamic-like system (for the classical velocity *V* and the probability *P*) with a macro-quantum potential depending on the concentration when *P* ∝ *c*,

(79) $$Q=-2{\text{D}}^{2}\frac{\Delta \sqrt{c}}{\sqrt{c}}$$

This hydrodynamics-like system also implicitly contains as a sub-part a standard diffusion Fokker-Planck equation with diffusion coefficient 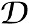 for the velocity *υ*_+_ (see Section 3). It is therefore possible to generalize the standard classical approach of biochemistry which often makes use of fluid equations, with or without diffusion terms (see, e.g., [61,62]).

Under the point of view of this third representation, the spontaneous transformation of a classical system into a quantum-like system through the action of fractality and irreversibility on small time scales manifests itself by the appearance of a quantum-type potential energy in addition to the standard classical energy balance. We have therefore suggested to search whether biological systems are characterized by such an additional potential energy [2]. This missing energy would be given by the above relation (Equation (79)) in terms of concentrations, and could be identified by performing a complete energy balance of biological systems, then by comparing it to the classically expected one.

However, we have also shown that the opposite of a quantum potential is a diffusion potential (Section 3.7). Therefore, in case of simple reversal of the sign of this potential energy, the self-organization properties of this quantum-like behavior would be immediately turned, not only into a weakly organized classical system, but even into an increasing entropy diffusing and disorganized system. We have tentatively suggested [2,8] that such a view may provide a renewed way of approach to the understanding of tumors, which are characterized, among many other features, by both energy affinity and morphological disorganization [63,64].

### 6.7. Nature of First Evolutionary Leaps

Another application of the scale relativity theory consists of applying it in the scale space itself. In this case, one obtains a Schrödinger equation acting in this space, and thus yielding peaks of probability for the scale values themselves. This yields a rough but already predictive model of the emergence of the cell structure and of the value of its typical scales.

Indeed, the three first events of species evolution are the appearance of prokaryot cells (about 3.5 Gyrs in the past), then of eukaryot cells (about 1.7 Gyr), then of the first multicellulars (about 1 Gyr). These three events correspond to three successive steps of organizational hierarchy.

Indeed, at the fundamental (‘vacuum’) level, one can expect the formation of a structure characterized by one length-scale (Figure 8). This particular scale is given by the peak of probability density. This problem is similar to that of a quantum particle in a box (but now it is a “box” in the space of scales), with the logarithms of the minimum scale λ*_m_* and maximum scale λ*_M_* playing the roles of the walls of the box. The fundamental level solution is well-known: it is a sinus curve whose peaks of probability lies in the middle of the box and vanishes on its walls. Since the “position” variable is here the logarithm of scales, this means that the fundamental level solution has a peak at a scale $\sqrt{\lambda_{m}\times \lambda_{M}}$.

What are the minimal and maximal possible scales? From a universal viewpoint, the extremal scales in nature are the Planck-length *l_P_* in the microscopic domain and the cosmic scale 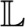 = Λ^−1/2^ given by the cosmological constant Λ in the macroscopic domain [8]. From the predicted and now observed value of the cosmological constant, one finds 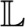/*l*_ℙ_ = 5.3 × 10^60^, so that the mid scale of the universe is at 2.3 × 10^30^ *l*_ℙ_ ≈ 40 μm.

Now, in a purely biological context, one would rather choose the minimal and maximal scales characterizing living systems. These are the atomic scale toward small scales (0.5 Angströms) and the scale of the largest animals like whales (about 10–30 m).It is remarkable that these values yield the same result for the peak of probability of the first structure of life, λ = 40 μm. This value is indeed a typical scale of living cells, in particular of the first ‘prokaryot’ cells appeared more than three Gyrs ago on Earth. Moreover, these prokaryotic first cells are, as described in this simple model, characterized by having only one hierarchical level of organization (monocellulars and no nucleus, see Figure 8).

The second level describes a system with two levels of organization, in agreement with the second step of evolution leading to eukaryots about 1.7 Gyrs ago (second event in Figure 8). One expects (in this very simplified model), that the scale of nuclei be smaller than the scale of prokaryots, itself smaller than the scale of eucaryots: this is indeed what is observed.

The following expected major evolutionary leap is a three organization level system, in agreement with the apparition of multicellular forms (animals, plants and fungi) about 1 Gyr ago (third event in Figure 8). It is also expected that the multicellular stage can be built only from eukaryotes, in agreement with the fact that the cells of multicellulars do have nuclei. More generally, it is noticeable that evolved organisms keep, inside their internal structure, the organization levels of the preceeding stages.

The following major leaps correspond to more complicated structures, then possibly to more complex functions (supporting structures such as exoskeletons, tetrapody, homeothermy, viviparity), but they are still characterized by fundamental changes in the number of organization levels. We also recall that a log-periodic acceleration has been found for the dates of these events [8,14,15,65,66], in agreement with the solutions of a “scale wave equation” (Equation (3)).

The first steps in the above model are based on spherical symmetry, but this symmetry is naturally broken at scales larger than 40 μm, since this is also the scale beyond which the gravitational force becomes larger than the van der Waals force. One therefore expects the evolutionary leaps that follow the apparition of multicellular systems to lead to more complicated structures (such as those of the Precambrian-Cambrian radiation), than can no longer be described by a single scale variable. This increase of complexity will be dealt with by extending this model to more general symmetries, boundary conditions and constraints.

### 6.8. Systems Biology and Multiscale Integration

We hope the scale relativity tools and methods to be also useful in the development of a systems biology framework [1,2,15]. In particular, such an approach would be in agreement with Noble's ‘biological relativity’ [67], according to which there is no privileged scale in living systems [68].

Now, one of the challenges of systems biology is the problem of multiscale integration [69]. The scale relativity theory allows to make new proposals for solving this problem. Indeed, its equations naturally yield solutions that describe multiscale structures, which are therefore spontaneously integrated. Let us illustrate this ability of scale relativity by giving a simple general example of the way the theory can be used to describe multiscale structuring.

The first step consists in defining the elements of description, which represents the smallest scale considered at the studied level. For example, at the cell level, these elementary structures could be intracellular “organelles”.

The second steps amounts to writing for these elementary “objects” an equation of dynamics which account for the fractality and irreversibility of their motion. As we have seen, such a motion equation written in fractal space can be integrated under the form of a macroscopic Schrödinger-type equation. This equation would no longer be based on the microscopic Planck constant, but on a macroscopic constant specific of the system under consideration (this constant can be related, e.g., to a diffusion coefficient). Its solutions are wave functions whose modulus squared gives the probability density of distribution of the initial “points” or elements.

**Figure 9:**
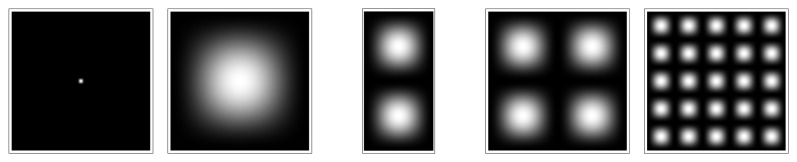
Multiscale integration in scale relativity. Elementary objects—at a given level of description (left figure)—are organized in terms of a finite structure described by a probability density distribution (second figure from the left). By increasing the energy, this structure spontaneously duplicates (third figure). New increases of energy lead to new duplications (fourth figure), then to a “tissue”-like organization (fifth figure—the scale of the figures is not conserved).

Actually, the solutions of such a Schrödinger-like equation are naturally multiscaled. It describes, in terms of peaks of probability density, a structuring of the “elementary” objects from which we started (e.g., organelle-like objects structuring at the larger scale cell-like level). As we have previously seen, while the vacuum state (lowest energy) usually describes one object (a single “cell”), excited states describe multiple objects (“tissue-like” level), each of which being often separated by zones of null density—therefore corresponding to infinite quantum potentials—which may represent “walls” (Figure 9). An increase of energy spontaneously generates a division of the single structure into a binary one, allowing one to obtain models of the growth of a “tissue” from a single “cell”.

A simple two-complementary-fluid model (describing, e.g., hydrophile/hydrophobe behavior) can easily be obtained, one of them showing probability peaks in the “cells” (and zero probability in the “wall”) while the other fluid peaks in the “walls” and have vanishing probability in the “cells”. This three-level multi-scale structure results from a general theorem of quantum mechanics (which remains true for the macroscopic Schrödinger regime considered here), according to which, for a one-dimensional discrete spectrum, the wave function corresponding to the (*n* + 1)th eigenvalue is zero *n* times [70]. A relevant feature for biological applications is also that these multi-scale structures, described in terms of stationary solutions of a Schrödinger-like equation, depend, not on initial conditions like in a classical deterministic approach, but on environment conditions (potential and boundary conditions).

Moreover, this scale relativity model involves not only the resulting structures themselves but also the way the system may jump from a two-level to a three-level hierarchical organization. Indeed, the solution of the time-dependent Schrödinger equation describes a spontaneous duplication when the energy of the system jumps from its fundamental state to the first excited state (see Section 6.4 and Figure 5 and Figure 9).

**Figure 10:**
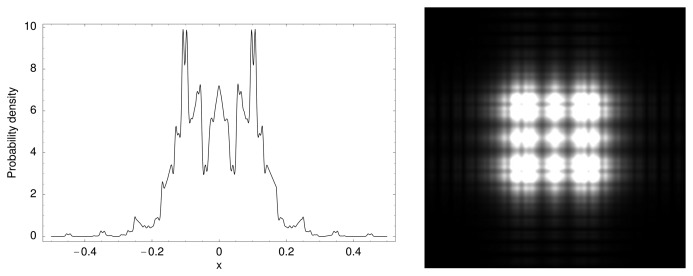
Fractal multiscale solutions of the Schrödinger equation. Left figure: one-dimensional solution in a box, in terms of position *x* for a given value of the time *t*. This solution reads $\psi (x,t)=(1/\pi )\sum_{n=-N}^{n=N}{\left(-1\right)}^{n}{\left(n+1/2\right)}^{-1}\exp\{i\pi [2x(n+1/2)-t{\left(n+1/2\right)}^{2}]\}$, with *N* → ∞ [21]. Finite resolution approximations of this solution can be constructed by taking finite values of *N*. Here the probability density |*ψ*|^2^ is drawn for *N* = 100 and *t* = 0.551. Right figure: fractal multiscale solution in a two dimensional box. It is constructed as a product *ψ*(*x*)*ψ*(*y*) of the one-dimensional solution given in the left figure.

One may even obtain solutions of the same equation organized on more than three levels, since it is known that fractal solutions of the Schrödinger equation do exist [8,21,22]. An example of such a fractal solution for the Schrödinger equation in a two-dimensional box is given in Figure 10.

Note that the resulting structures are not only qualitative, but also quantitative, since the relative sizes of the various embedded levels can be derived from the theoretical description. Finally, such a “tissue” of individual “cells” can be inserted in a growth equation which will itself take a Schrödinger form. Its solutions yield a new, larger level of organization, such as the flower-like structure of Figure 3. Then the matching conditions between the small scale and large scale solutions (wave functions) allow to connect the constants of these two equations, and therefore the quantitative scales of their solutions.

## 7. Conclusions

The theory of scale relativity, thanks to it accounting for the fractal geometry of a system at a profound level, is particularly adapted to the construction and development of a theoretical biology. In its framework, the description of living systems is no longer strictly deterministic. It supports the use of statistical and probabilistic tools in biology, for example as concerns the expression of genes [71,72].

However, it also suggests to go beyond ordinary probabilities, since the description tool becomes a quantum-like (macroscopic) wave function, which is the solution of a generalized Schrödinger equation. This involves a probability density such that *P* = |*ψ*|^2^, but also phases which are built from the velocity field of potential trajectories and yield possible interferences.

Such a Schrödinger (or non-linear Schrödinger) form of motion equations can be obtained in at least two ways. One way is through the fractality of the biological medium, which is now validated at several scales of living systems, for example in cell walls [73]. Another way is through the emergence of macroscopic quantum-type potentials, which could be an advantageous character acquired from evolution and selection.

In this framework, one therefore expects a fundamentally wave-like, and often quantized, character of numerous processes implemented in living systems. In the present contribution, we have concentrated on the theoretical aspect of the scale relativity approach, then we have given some examples of applications to some biological processes and functions.

Several properties that are considered to be specific to biological systems, such as self-organization, morphogenesis, ability of duplicating, reproducing and branching, confinement, multi-scale structuration and integration are naturally obtained in such an approach [1,2]. The implementation of this type of process in new technological devices involving intelligent feedback loops and quantum-type potentials could also lead to the emergence of a new form of ‘artificial life’.
